# Assessing knowledge of sickle cell disease and health beliefs on premarital genetic screening among healthcare trainees at a tertiary institution: A cross‐sectional study

**DOI:** 10.1002/hsr2.1128

**Published:** 2023-02-24

**Authors:** William Kwaku Agbozo, Ernest Amanor, Eugene Owusu Acheampong, Brenda Kotei, Leslie Nii Attoh, David Yeboah, Prince Odonkor, Seth Obiri, Alexander Kwarteng, Amma Larbi

**Affiliations:** ^1^ Department of Physician Assistantship Central University Miotso Ghana; ^2^ West African Genetic Medicine Centre (WAGMC) University of Ghana Legon‐Accra Ghana; ^3^ Department of Biochemistry and Biotechnology, College of Science Kwame Nkrumah University of Science and Technology Kumasi Ghana; ^4^ Department of Epidemiology and Biostatistics Kwame Nkrumah University of Science and Technology Kumasi Ghana; ^5^ Department of Nursing Central University Miotso Ghana; ^6^ Kumasi Centre for Collaborative Research in Tropical Medicine Kwame Nkrumah University of Science and Technology Kumasi Ghana

**Keywords:** genetic counseling, premarital, sickle cell disease, sickle cell trait

## Abstract

**Background:**

The uptake of sickle cell trait (SCT) test is challenged by several factors. A community of healthcare professionals educating the public to undergo screening is critical in reducing the disease burden. We investigated knowledge and attitude towards premarital SCT screening among healthcare trainee students who are the next generation of healthcare practitioners.

**Methods:**

A cross‐sectional design was employed, and quantitative data were collected from 451 female students pursuing healthcare programs at a tertiary institution in Ghana. Descriptive, bivariate, and multivariate logistic regression analysis was performed.

**Results:**

More than half of the participants were 20–24 years (54.55%) and had good knowledge (71.18%) about sickle cell disease (SCD). Age and school or social media as sources of information were significantly associated with good knowledge about SCD. Students between the age 20–24 (adjusted odds ratio [AOR] = 2.54, confidence interval [CI] = 1.30–4.97) and knowledge (AOR = 2.19, CI = 1.41–3.39) were 3 times and 2 times more likely to have a positive perception about SCD severity. Students who have SCT (AOR = 5.16, CI = 2.46–10.82), whose source of information was family member/friends (AOR = 2.83, CI = 1.44–5.59) and social media (AOR = 4.59, CI = 2.09–10.12) were 5 times, 2 times and 5 times likely to have a positive perception about the susceptibility of SCD. Students whose source of information is school (AOR = 2.06, CI = 1.11–3.81) and who have good knowledge of SCD (AOR = 2.25, CI = 1.44–3.52) were 2 times more likely to have a positive perception about the benefits of testing. Students with SCT (AOR = 2.64, CI = 1.36–5.13) and source of information was social media (AOR = 3.01, CI = 1.36–6.64) were about 3 times more likely to have a positive perception about the barriers to testing.

**Conclusion:**

Our data shows that high level of SCD knowledge influences positive perceptions about the severity of SCD, the benefits and relatively low barriers to SCT or SCD testing and genetic counseling. Dissemination of SCT, SCD and premarital genetic counseling education should be intensified especially in schools.

## 1 INTRODUCTION

Sickle cell disease (SCD) is a genetic disorder that is inherited at birth from parents with sickle cell trait (SCT). In the tropics, SCD is commonly present in the severe homozygous SS (HbSS), heterozygous SC (HbSC), and the beta‐zero or beta‐plus thalassemia (HbSβ°/HbSβ^+^ thalassemia).^1^, ^2^ Globally, it is estimated that nearly 5% of the world's population have SCT, and almost 300,000 babies are born with SCD annually.^3^, ^4^ Ghana significantly contributes to this global prevalence, with about 2% (approximately 15,000) of all newborns having SCD, and more than half have the severe form of the disease.^5^ The disease is characterized by several symptoms and complications, such as vaso‐occlusive pain episodes, hemolysis, anemia, acute chest syndrome, stroke, and dactylitis.^2^, ^6^ Due to the severity of the disease, patients experience poor quality of life, and caregivers are significantly burdened.^7^, ^8^

Early diagnosis of SCD coupled with comprehensive care has been effective in newborn morbidity and mortality reduction. The newborn screening program has been initiated in Africa and Ghana, and this has been one of the successful forms of population screening for SCD improving newborn survival and related health outcomes.^9^, ^10^, ^11^ Since it was started in the mid‐1990s in Ghana, the program has proven to be a cost‐effective intervention with over 170,000 newborns screened and most maintaining necessary follow‐up.^9^ The primary purpose of the newborn screening program is to identify newborns with SCD by taking blood through a heel prick in a “spot” on a special paper for haemoglobin electrophoresis.^11^, ^12^, ^13^ Subsequently, penicillin prophylaxis which helps to substantially reduce the incidence of pneumococcal sepsis during infancy is initiated.^11^, ^12^, ^13^ Further, during the screening for SCD, babies with SCT are identified, and this affords parents the opportunity to know their SCT status. Therefore, parents who may be at risk of giving birth to an SCD child can be counseled on the consequences of future pregnancies. However, a recent study conducted in Ghana has shown that the program needs a revamp. In a population of 354 SCD patients, only 5.5% of the patients were diagnosed by newborn screening, whilst 86.9% were diagnosed by non‐newborn screening (including hospitalization and medical history), and 7.6% were diagnosed based on family history.^9^ This may imply that many parents might not be privy to their SCT status and seek counseling before the next pregnancy.

In Ghana, simple blood test, haemoglobin electrophoresis and other new technologies for the detection of SCT and genotype are available in all regional hospitals and some district and private hospitals across the country. The capacity of genetic counselors has also improved in Africa and Ghana in recent times,^14^, ^15^ and this offers families, parents or couples an opportunity to seek for genetic counseling before getting married and procreating. However, there are no definitive public screening policies, making identification of the SCT an individual decision among the populace. The uptake of genetic counseling has been challenged by several factors, including knowledge level of SCD, health beliefs, cultural beliefs, and economic factors.^15^ These factors reduce the interest and support of people for SCT testing and genetic counseling. Hence. to improve SCT testing and uptake of genetic counseling most importantly before marriage and procreation, it is imperative to understand and tackle these barriers. This can be achieved by employing the health belief model (HBM). The HBM is a theory of behavioral change that over the years has been employed to evaluate the compliance of medical regimens in various healthcare disciplines.^16^ The principle of this model is that the acceptance and compliance of an intervention could be enhanced through an individual's perceived susceptibility to the condition; an individual's perceived seriousness of the condition; an individual's perceived benefit of the intervention; and an individual's perceived barriers to the intervention.^16^ This can then inform the design of educational and counseling strategies. Using HBM, a recent study by Gustafson et al.^17^ has demonstrated high average knowledge level was associated with a high perception of the severity and benefit of SCD screening and genetic counseling. On the contrary, some of the participants do not believe that they are at risk of giving birth to a child with the disease. This may affirm that increased knowledge and understanding of SCD may enhance the acceptance and participation in SCT screening and premarital genetic counseling.

Built into the importance of enhanced education and counseling towards SCT screening is the need to have a community of healthcare professionals who are both equipped with knowledge in disease genetics and understand the need to rightly guide the larger population towards positive premarital genetic screening attitudes. The belief and attitude of healthcare professionals is a major contributing factor to decisions towards SCT screening. However, attitude of health care professionals towards premarital SCT screening is largely unexplored in research. While few studies emerging from the developed countries reports on the subject, a study from Ghana is yet to be reported. Therefore, this study sought to investigate knowledge on SCD and health beliefs with regards to attitude towards premarital SCT screening and genetic counseling among healthcare trainee students who are the next generation of healthcare practitioners.

## 2 MATERIALS AND METHODS

### 2.1 Study area

The study was conducted at the Central University, Ghana, among female students pursuing all heath related courses offered by the University. The Central University is a private institution of higher learning located in Mistso, the capital region of Ghana, and lies at the coordinates 5.5543414, −0.2323346. The university has a student population of about 8400 and offers courses in Politics, History and Law, Business, Finance and Economics, Geography and Agriculture, Humanities and Social Sciences, Arts, Design and Music, Engineering and Technology, Health and Medicine, and Media and Communication.

### 2.2 Data

The study employed a cross‐sectional design with a quantitative method of data collection. Data captured socio‐demographic characteristics, knowledge of SCD, and health beliefs about SCD. The HBM was adapted to collect data on health beliefs. Ethical clearance (Refs: CU‐IRB 2020/21) was obtained from the Committee of Ethical Clearance and Institutional Review Board, of the Central University (CU‐IRB), Ghana.

### 2.3 Methods

Female students pursuing one of the healthcare programs the University offers (i.e., Pharmacy, Nursing, and Physician Assistantship) were approached face‐to‐face in their lecture rooms. A 100% consent to participate was recorded after the purpose of the study was be explained to them. Participants were then selected by a multistage sampling approach, where participants were selected from 12 classes (representing level 100–400 classes for the 3 healthcare programs the University offers) by simple random sampling. Female students belonging to each class balloted by drawing with replacement a piece of paper with the inscription “Yes‐recruited” and “No‐not recruited”. A total of 451 study participants out of a total population (*N*) = 900 female students pursuing one of the healthcare programs were recruited. Minimum sample size of was determined using sample size calculation, $n=[z^{2}\times p\times (1\;–p)/\;e^{2}]/[1\;+(z^{2}\;\times p\times (1\;–p)/z^{e}\;\times N)]$ and based on the assumed prevalence on knowledge level about SCD i.e., 58.1%, reported by Ugwu (2016) who assessed the awareness, knowledge, and attitude among undergraduate students of a tertiary educational institution. Z score (z) was set at 1.96 (95% confidence interval [CI]), margin of error (e) at 5% and standard deviation (p) of 0.5. A well‐structured questionnaire was employed to interview the study participants in‐person.

### 2.4 Statistics

The data were entered into an MS Excel, cleaned, and imported into STATA v.16 for analysis. Descriptive analysis was performed, expressed in frequencies and percentages, and presented in tables and figures. Further bivariate and multivariate logistic regression analyses were performed to elucidate factors associated with knowledge of SCD and perceptions about premarital genetic counseling among the study participants. Statistical significance was considered at *p* < 0.05.

### 2.5 Health belief model

The HBM is a theory of behavioral change that consists of seven components including perceived threat, perceived susceptibility, perceived severity, perceived benefits, perceived barriers, cues to action and self‐efficacy. However, in this study the perceived susceptibility, perceived severity, perceived benefits, perceived barriers were assessed among the study participants. The perceive susceptibility component emphasizes an individual's perception of the risk of developing a condition. For instance, if an individual feels susceptible to SCD and more likely to accept SCT screening as opposed to others who do not believe in this. The perceived severity component explains that a condition such as SCD could cause a severe form of illness and, therefore, this could influence one's decision to accept SCT screening. The perceived benefits refers to an individual's understanding of the effectiveness of available interventions to reduce the threat of illness or disease. Thus, if SCT screening is perceived to be beneficial, an individual is likely to accept it. In the perceived barriers component, an individual perceives the obstacles to acceptance of SCT screening. There is wide variation in a person's feelings of barriers, or impediments, which lead to a cost/benefit analysis. The person weighs the effectiveness of the actions against the perceptions that it may, be dangerous (e.g., side effects), unpleasant (e.g., painful), time‐consuming, or inconvenient, Distance (Hard to reach communities etc.).


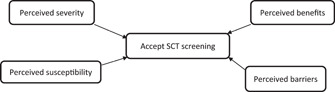


## 3 MEASUREMENT

### 3.1 Dependent/outcome variables

The outcome variable “Knowledge” was measured binary using “good knowledge” defined as a knowledge score equal or above the median knowledge score, and “poor knowledge” defined as a knowledge score below the median knowledge score. Moreover, the outcome variable “Perception” was measured binary using “positive perception” defined as a perception score equal or above the median/mean perception score and “negative perception” defined as a perception score below the median/mean perception score. Perception score was computed for the severity model, susceptibility model, barrier model, and benefit model.

### 3.2 Independent/predictor variables

The predictor variables for the knowledge outcome variable include age, marital status, tested for SCT, found to have SCT, and sources of information about SCD. On the other hand, the predictor variables for the perception outcome variable included age, and marital status, tested for SCT, found to have SCT, sources of information, and knowledge on SCD.

## 4 RESULTS

### 4.1 Socio‐demographic characteristics

**Figure 1:**
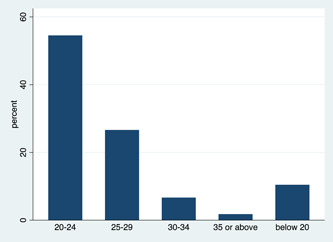
Distribution of study participants by age.

**Figure 2:**
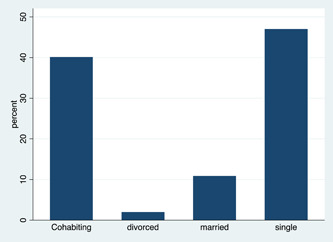
Distribution of study participants by marital status.

From Table 1, a total of 451 participants were interviewed of which more than two thirds (81.16%) were within the age range of 20–29 (Figure 1) and were yet to marry (87.14%) (Figure 2). We sought to identify participants who knew their SCT and it was revealed that about 64% have not tested for SCT. Moreover, only about 11% of the participants have SCT.

**Table 1:** Socio‐demographic characteristics of study participants.

Variables	Frequency	Percentage (%)
Age	*N* = 451	
Below 20	47	10.42
20–24	246	54.55
25–29	120	26.61
30–34	30	6.65
35 and above	8	10.42
Marital status	*N* = 451	
Single	212	47.01
Dating	181	40.13
Married	49	10.86
Divorced	9	2.00
Tested for sickle cell trait	*N* = 451	
No	289	64.08
Yes	162	35.92
Found to have sickle cell trait	*N* = 451	
No	402	89.14
Yes	49	10.86

### 4.2 Knowledge level of SCD

The knowledge of the study participants on SCD was assessed and the median knowledge score was 5 with an interquartile range of 4–7. The majority (71.18%) of the participants had good knowledge about SCD. Moreover, the school (38.58%) was the common source of information about SCD, followed by a health professional (18.63%) and the family (15.74%) as shown in Table 2.

**Table 2:** Knowledge of sickle cell disease among study participants.

Variable	Frequency	Percentage
Outcome of knowledge	*N* = 451	
Median (interquartile range)	5 (4–7)	
Poor	130	28.82
Good	321	71.18
Sources of information about sickle cell disease	*N* = 451	
Hospital/health professional	84	18.63
Family member/friend	71	15.74
At school	174	38.58
Social media	54	11.97
Television/radio program	65	14.41
Other	3	0.67

### 4.3 Perceived health beliefs about genetic testing of SCT

**Figure 3:**
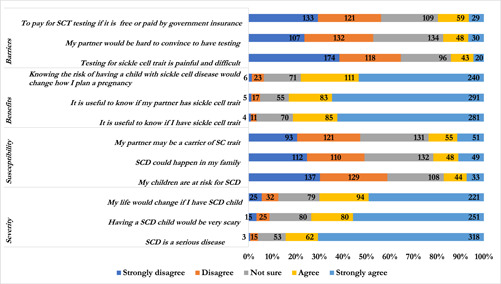
Health beliefs regarding perceived severity, susceptibility, benefits, and barriers to genetic testing for sickle cell trait.

The perceived health beliefs about genetic testing for SCT were assessed based on four models. The median severity score was 14, and 60.75% had a positive perception of the severity of SCD (Table 3). Further, the majority strongly agree that SCD is a serious disease (70.51%), having an SCD child would be very scary (55.65%), and my life would change if I have SCD child (49.00%) as shown in Figure 3. On the susceptibility to SCD, about 30% of the participants strongly disagree that their children are at risk for SCD, 29.7% are not sure whether SCD could happen in the family whilst 29.05% are not sure their partner may be a carrier of SCT (Figure 3). Further, the mean susceptibility score was 7.60, and more than half of the participants had a negative perception of the susceptibility of SCD, as shown in Table 3. Assessing participant's perceived benefits, the median benefits score reported was 14, and 65.41% had a positive perception of benefits of testing (Table 3). Most of them strongly agreed to the statement that “it is useful to know if I have SCT” (62.31%), “it is useful to know if my partner has SCT” (64.52%), and “knowing the risk of having a child with cell” (53.22%) (Figure 3). On the barriers to testing for SCT, the mean barrier score was 7.02, and 51.88% of the participants had a positive perception of the barriers to SCT testing (Table 3). The majority of the participants strongly disagreed testing for SCT is painful and difficult (38.58%), 29.71% are not sure of the statement “my partner would be hard to convince to have testing” and about 30% strongly disagree to pay for SCT testing if it is free or paid by government insurance (Figure 3).

**Table 3:** Perceptions on premarital genetic testing among study participants.

Variables	Perception	
	Negative	Positive
Severity	177 (39.25)	274 (60.75)
	14 (15–11)^a^	
Susceptibility	230 (51.00)	221 (49.00)
	7.60 (±3.07)^b^	
Benefits	156 (34.59)	295 (65.41)
	14 (11–15)^a^	
Barriers	217 (48.12)	234 (51.88)
	7.02 (±2.87)^b^	

a Median (interquartile range).

b Mean (standard deviation).

### 4.4 Factors associated with good knowledge level on SCD

The crude odds ratio was computed, which revealed that age 20–24 and 25–29 was significantly associated with good knowledge about SCD. Also, social media as the source of information about SCD was significantly associated with good knowledge. The above data is described in Table 4.

**Table 4:** Factors influencing the knowledge level of sickle cell disease among study participants.

Variable	Knowledge level
	COR (95% CI)
Age	
Below 20	Ref
20–24	4.20 (2.20–8.03)***
25–29	3.01 (1.50–6.04)**
30–34	2.48 (0.96–6.42)
35 and above	2.06 (0.44–9.65)
Marital status	
Divorced	Ref
Dating	1.61 (0.39–6.69)
Married	1.25 (0.27–5.71)
Single	1.01 (0.25–4.18)
Tested for sickle cell trait	
No	Ref
Yes	1.52 (0.98–2.37)
Found to have sickle cell trait	
No	Ref
Yes	1.91 (0.90–4.07)
Sources of information about sickle cell disease	
Hospital/health professional	Ref
At school	1.01 (0.55–1.87)
Family member/friend	0.70 (0.34–1.42)
Social media	0.42 (0.20–0.88)*
Television/radio program	0.61 (0.30–1.25)
Other	0.16 (0.01–1.82)

Abbreviations: CI, confidence interval; COR, crude odds ratio; Ref, reference group.

* *p* < 0.05

** *p* < 0.005

*** *p* < 0.001.

### 4.5 Factors influencing the positive perceptions about premarital genetic testing

To determine the factors that influence positive perceptions about premarital genetic testing of SCD; age, marital status, tested for SCT, found to have SCT, sources of information and knowledge of SCD were adjusted. The analysis showed that participants between the age 20–24 (adjusted odds ratio [AOR] = 2.54, CI = 1.30–4.97) were about 3 times more likely to a have positive perception of severity whilst those between ages 30–34 (AOR = 0.22, CI = 0.07–0.69) were 78% less likely to have a positive perception about the severity of SCD than their counterparts. Participants dating (AOR = 6.86, CI = 1.30–36.34) and with good knowledge (AOR = 2.19, CI = 1.41–3.39) were about 7 times and 2 times more likely to have positive perceptions about the severity of SCD, respectively. However, participants whose source of information was social media (AOR = 0.36, CI = 0.17–0.76) and found to have SCT (AOR = 0.36, CI = 0.18–0.74) are 64% less likely to have a positive perception about the severity of SCD. Age; found to have SCT, source of information and knowledge of SCD was adjusted for susceptibility to SCD, benefits of testing, and barriers to testing. It was revealed that participants who have SCT (AOR = 5.16, CI = 2.46–10.82), whose source of information was family members/friends (AOR = 2.83, CI = 1.44–5.59) and social media (AOR = 4.59, CI = 2.09–10.12) were 5 times, 2 times and 5 times likely to have a positive perception about the susceptibility of SCD respectively. However, the participants with good knowledge (AOR = 0.60, CI = 0.39–0.92) were 40% less likely to have a positive perception of the susceptibility of SCD. On the benefits of testing, participants between the age 30–34 (AOR = 0.37, CI = 0.14–0.99) and who has SCT (AOR = 0.34, CI = 0.18–0.64) were 63% and 66% less likely to have positive perceptions about the benefits of testing respectively. Nonetheless, those whose source of information is school (AOR = 2.06, CI = 1.11–3.81) and who have good knowledge of SCD (AOR = 2.25, CI = 1.44–3.52) were 2 times more likely to have a positive perception about the benefits of testing. On the other hand, participants between the ages 20–24 (AOR = 0.38, CI = 0.19–0.78) and with good knowledge of SCD (AOR = 0.38, CI = 0.25–0.59) were 62% less likely to have a positive perception about the barriers to testing. However, participants who have SCT (AOR = 2.64, CI = 1.36–5.13) and a source of information was social media (AOR = 3.01, CI = 1.36–6.64) were about 3 times more likely to have a positive perception about the barriers to testing. The above data is described in Table 5.

**Table 5:** Factors Influencing the positive perceptions about premarital genetic testing among study participants.

Variable	Severity		Susceptibility		Benefits		Barriers	
	COR (95% CI)	AOR (95% CI)	COR (95% CI)	AOR (95% CI)	COR (95% CI)	AOR (95% CI)	COR (95% CI)	AOR (95% CI)
Age								
Below 20	Ref	Ref	Ref	Ref	Ref	Ref	Ref	Ref
20–24	3.38 (1.78–6.41)**	2.54 (1.30–4.97)*	0.61 (0.33–1.15)	0.82 (0.42–1.59)	1.80 (0.93–3.49)	1.24 (0.61–2.51)	0.28 (0.14–0.55)***	0.38 (0.19–0.78)*
25–29	1.42 (0.72–2.79)	1.20 (0.59–2.48)	0.92 (0.47–1.82)	0.91 (0.45–1.87)	0.61 (0.30–1.21)	0.52 (0.25–1.09)	0.59 (0.28–1.24)	0.66 (0.30–1.42)
30–34	0.25 (0.08–0.76)*	0.22 (0.07–0.69)*	1.40 (0.55–3.57)	1.21 (0.45–3.26)	0.38 (0.15–0.97)*	0.37 (0.14–0.99)*	0.66 (0.25–1.76)	0.63 (0.22–1.75)
35 and above	1.24 (0.28–5.55)	1.08 (0.22–5.26)	2.42 (0.44244–13.27)	2.65 (0.45–15.70)	0.57 (0.13–2.56)	0.53 (0.11–2.65)	0.64 (0.13–3.06)	0.66 (0.13–3.50)
Marital status								
Divorced	Ref	Ref	Ref		Ref		Ref	
Dating	8.68 (1.75–43.18)**	6.86 (1.30–36.34)*	0.41 (0.09–1.67)		2.86 (0.74–11.07)		0.40 (0.10–1.63)	
Married	1.14 (0.21–6.22)	1.35 (0.24–7.73)	1.54 (0.33–7.13)		0.61 (0.14–2.57)		1.39 (0.30–6.36)	
Single	5.66 (1.15–27.92)*	4.54 (0.84–24.50)	0.42 (0.10–1.73)		2.96 (0.77–11.37)		0.56 (0.14–2.30)	
Tested for sickle cell trait								
No	Ref	Ref	Ref		Ref		Ref	
Yes	0.61 (0.42–0.90)*	0.75 (0.47–1.19)	1.24 (0.85–1.83)		1.14 (0.76–1.71)		0.71 (0.48–1.04)	
Found to have sickle cell trait								
No	Ref	Ref	Ref	Ref	Ref	Ref	Ref	Ref
Yes	0.30 (0.16–0.56)***	0.36 (0.18–0.74)**	4.71 (2.29–9.70)***	5.16 (2.46–10.82)***	0.35 (0.19–0.64)**	0.34 (0.18–0.64)**	2.29 (1.21–4.33)*	2.64 (1.36–5.13)**
Sources of information about sickle cell disease								
Hospital/health professional	Ref	Ref	Ref	Ref	ref	Ref	Ref	Ref
At school	1.75 (1.01–3.03)	1.49 (0.83–2.66)	0.79 (0.47–1.35)	0.96 (0.55–1.68)	2.34 (1.30–4.22)*	2.06 (1.11–3.81)*	0.54 (0.32–0.92)*	0.58 (0.34–1.00)
Family member/friend	0.67 (0.35–1.26)	0.55 (0.28–1.09)	2.42 (1.27–4.64)*	2.83 (1.44–5.59)**	0.61 (0.32–1.16)	0.09 (0.28–1.09)	1.55 (0.82–2.95)	1.59 (0.82–3.09)
Social media	0.35 (0.17–0.71)**	0.36 (0.17–0.76)*	4.42 (2.07–944)***	4.59 (2.09–10.12)***	0.42 (0.21–0.85)*	0.48 (0.23–0.99)*	3.34 (1.54–7.22)**	3.01 (1.36–6.64)*
Television/radio program	1.04 (0.53–2.01)	0.96 (0.47–1.96)	1.54 (0.80–2.95)	1.67 (0.84–3.30)	0.62 (0.32–1.19)	0.65 (0.32–1.30)	1.53 (0.79–2.95)	1.49 (0.76–2.94)
Other	0.32 (0.023–3.71)	0.54 (0.05–6.51	1.00	1.00	0.26 (0.02–3.03)	0.44 (0.04–5.23)	1.91 (0.17–21.84)	1.44 (0.12–17.79)
Knowledge of SCD								
Poor	Ref	Ref	Ref	Ref	Ref	Ref	Ref	
Good	2.04 (1.35–3.09)**	2.19 (1.41–3.39)***	0.61 (0.41–0.92)*	0.60 (0.39–0.92)*	2.10 (1.38–3.20)**	2.25 (1.44–3.52)***	0.38 (0.25–0.58)***	0.38 (0.25–0.59)***

Abbreviations: AOR, adjusted odds ratio; CI, confidence interval; COR, crude odds ratio; Ref, reference group.

* *p* < 0.05

** *p* < 0.005

*** *p* < 0.001.

## 5 DISCUSSION

The psychological, emotional, economic and painful burden exerted by SCD condition on its patients and their families has increased the need for education and creating awareness of the condition over the past two decades worldwide.^18^ Individuals’ who are well informed on SCD and its socioeconomic burdens, accept to screen for SCT and make good reproductive decisions against the risk of SCD births. In Ghana most people rely on the responsibility of the healthcare practitioner to be educated and be guided on their health decisions. Ghana has limited information on the interest and attitude of health care professionals towards encouraging premarital SCT screening and genetic counseling. We were therefore motivated to explore this gab among healthcare trainees who are the next generation of healthcare practitioners. In this study, we solicited views from young female healthcare trainee students with majority (87.13%) of them about to experience motherhood and be faced with making reproductive health decisions. Women experience direct consequences of negative attitude towards reproductive choices. However, women are constrained by social, legal, religious, cultural impediments as well as family power dynamics to autonomously make reproductive decisions. Studies such as ours, reporting on women's independent reproductive beliefs and choices is needed to promote women's right to reproductive decision‐making. Findings from our participants who are the next generation of healthcare practitioners will help predict the influence they will have on the larger public female population who need be encouraged to be actively involved in reproductive choices.

A greater proportion of our study participants had good knowledge of SCD. This was consistent with findings in Ghana and Nigeria, where high knowledge of SCD was documented among tertiary students.^19^, ^20^, ^21^ The increased publicity most likely translates into increased awareness and knowledge among individuals which could explain the similarities in the findings of the studies. Also, we found that students between 20 and 29 years of age are more likely to have a good knowledge level of SCD. Moreover, marital status did not predict knowledge of SCD. Similarly, Boadu^20^ and Abubakar et al.^22^ revealed no statistically significant association between knowledge of SCD and marital status. Unlike our findings, Petrou et al.^23^ and Alkalbani et al.^24^ found that marital status was significantly associated with knowledge among their study participants. However, the populations employed by these studies were prospective couples and those married, which could account for the differences in the findings with regard to the current study. Furthermore, there was no statistically significant association between the age of study participants and knowledge of SCD,^23^, ^24^ and this contradicts our findings. The noticeable variations in the study finding could be attributed to the disparities in the age distributions of these two studies. Good knowledge of SCD increases the likelihood of perceived severity and benefits of premarital genetic testing for SCD. Gustafson et al. also found that good SCD knowledge significantly predicted the perceived severity of the disease among their study participants.^17^ Nevertheless, participants with good knowledge perceived low barriers in premarital testing for SCD, and this conforms with our findings.^17^ We recommend the implementation of more studies to validate these findings.

In addition, this study showed that the school system plays a significant role in the dissemination of information on SCD. Here, we found that a significant number of these students sourced information on the condition from health facilities or health professionals, family/friends, as well as media outlets such as television/radio programs and social media. Similarly, Isah et al.^19^ and Ugwu^21^; reported the school system to be a frequent source of information on SCD among students in Nigeria. These findings reveal the efforts of educational institutions in the fight against SCD. Therefore, it is important that policymakers capitalize on this resource to widen the educational scope on SCD. Over the years, the media has contributed immensely to the sensitization of people about SCD and premarital genetic counseling.^15^, ^25^, ^26^ However, dissemination of SCT or SCD and premarital counseling information on social media could prove to be a game changer. This is because majority of the youth spend a significant part of their time on social media^27^, ^28^ and, therefore, there is higher likelihood of becoming privy to this information to influence their health beliefs about the disease.

The health belief of the students on SCT or SCD testing and the need for premarital genetic counseling was assessed on four constructs of the HBM; perceived severity, perceived susceptibility, perceived benefits and perceived barriers.^16^, ^29^ On the perceived severity of SCD, we documented higher scores of perceived severities of SCD among participants. Further, most of our study participants expressed a perceived change of lifestyle if they are to give birth to a child with SCD. Similarly, the majority perceived SCD to be a serious health condition, and it is very scary. This resonates with the findings of Smith and Mercado‐Sierra,^30^ Gustafson et al.,^17^ and Al‐Azri et al.,^31^ who reported high scores of perceived severity of SCD. They also reported that more than a third quarter of these participants strongly believed SCD to be a serious disease, having a child with SCD would be a life‐changing event, and having a child with SCD would be scary.^17^, ^30^, ^31^ Our study also found that participants within the age group of 20–24 years independently increased the probability of perceiving SCD as a severe health condition. Similarly, being in relationship(dating) and having good knowledge of SCD increased the chance of perceiving SCD as a severe health condition. However, there were reduced odds of perceiving disease as a severe health condition among participants with the sickle trait. Unlike our findings, Gustafson et al.^17^ and Al‐Azri et al.^31^ did not find an association between the perceived severity of SCD and the demographic variables of the participants. Advancement in science over the past decade has aided adequate management of SCD pain episodes, relieving symptoms, and prevention of complications through medication and other medical procedures.^32^, ^33^ These milestones could explain the reduced perception of the severity of the condition. These findings need validation, and we recommend more studies that will investigate the association between the perceived severity of SCD among students.

The current study recorded a relatively lower score of perceived susceptibility to SCD among participants. The majority of our participants disagreed that their children will be at risk of SCD. Moreover, a significant proportion disagreed that their partners might be carriers of SC trait and the possibility that SCD could happen in their family. This contradicts the findings of Al‐Azri et al.,^31^ who documented that majority of their study did not agree nor disagree with these statements. Also, Smith and Mercado‐Sierra^30^ showed that 7% of their participants believed their children were at risk of SCD, 10% agreed that their partners could be carriers of SC traits, and 21% believed SCD could be present in their family. Further, Smith and Mercado‐Sierra^30^ also documented that the increasing age of the participants was significantly associated with reduced perception of SCD susceptibility among the study participants. These findings were also consistent with the findings of Gustafson et al.,^17^ who documented that perceived susceptibility of SCD significantly correlates with age, and this resonates with our findings. This may be attributed to the fact that the participants may not have been privy to the symptoms of SCD and may have never experienced these symptoms, hence the reduced perception of SCD susceptibility.

We found lower scores of perceived barriers to SCD genetic testing among our study participants. The majority of these participants disagreed with difficulty in convincing their partners in taking the test and thier perception of SCT test being painful and difficult. This was consistent with the findings of similar studies conducted in Ghana, where the participants perceived relatively low barriers to SCT testing.^15^, ^25^, ^34^ Moreover, Gustafson et al validate these findings.^17^ However, Smith and Mercado‐Sierra reported a higher mean score (21.81) for the perceived barrier to SCT testing.^30^ Also, a higher proportion of their participants revealed their unwillingness to pay for SCD testing if it is not incorporated into their subscribed insurance package.^30^ Similar to our findings, the majority of their participants did not perceive difficulty in convincing their partners to take the test nor experiencing pain due to the procedure involved in testing.^30^ Nonetheless, contrasted with the findings of Al‐Azri et al.^31^ Increased knowledge of the SCD and the consequences of having children with the condition necessitates necessary action to avoid being in the bracket of parents with children with SCD. This is likely to encourage testing for SCD before marriage regardless of the barriers.

Further, the participants positively perceived the benefits of premarital testing for SCD. Most participants perceived the need to know the genetic trait of thier partner before marriage. As reported in another study among students, a positive perception of the benefit of testing is likely to encourage participants to test before making the decision to get married.^17^, ^30^ We found that participants within the age group 30–34 years decreased the likelihood of having a positive perception of the benefit of premarital genetic testing. Similarly, another study reported decreased perceived benefits among students ≤35.^31^ This observation could be due to the fact that these individuals within this age group may have been married at the time the survey was being conducted hence do not perceive the benefits of screening for SCD or SCT and seeking genetic counseling since they are already married.

## 6 CONCLUSION

Knowledge of SCD was significantly high among the trainees. Age and source of information were significant determinants of the students’ knowledge level of SCD. Tertiary education plays a crucial role in influencing students’ attitude towards premarital SCT screening and genetic counseling. Dissemination of SCD and premarital SCT screening should target the media (particularly social media since majority of the youth spend much time on social media), health professionals and family/friends. Most of the students showed positive perceptions about the severity of SCD, the benefits and relatively low barriers to SCT or SCD testing and genetic counseling. Our findings suggests that our study participants will be good agents towards SCD awareness creation. However, most of the students do not believe they have a high risk of having a child with SCD and there is a need to intensify education among students on their perceptions on the susceptibility of SCD.

## AUTHOR CONTRIBUTIONS

**William Kwaku Agbozo**: conceptualization; investigation; methodology; resources; supervision; writing—review & editing. **Ernest Amanor**: formal analysis; writing—original draft; writing—review & editing. **Eugene Owusu Acheampong**: formal analysis; writing—original draft; writing—review & editing. **Brenda Kotei**: data curation; investigation; writing—review & editing. **Leslie Nii Attoh**: data curation; investigation; writing—review & editing. **David Yeboah**: investigation; writing—review & editing. **Prince Odonkor**: investigation; writing—review & editing. **Seth Obiri**: investigation; writing—review & editing. **Alexander Kwarteng**: writing—original draft; writing—review & editing. **Amma Larbi**: project administration; supervision; writing—original draft; writing—review & editing.

## CONFLICT OF INTEREST STATEMENT

The authors declare no conflicts of interest.

## TRANSPARENCY STATEMENT

The lead author William Kwaku Agbozo, Amma Larbi affirms that this manuscript is an honest, accurate, and transparent account of the study being reported; that no important aspects of the study have been omitted; and that any discrepancies from the study as planned (and, if relevant, registered) have been explained.

## Data Availability

The data that support the findings of this study are available from the corresponding author upon reasonable request.
